# Supplementary material for: Patterns of Intron Gain and Loss in Fungi
Source: PLoS Biol. 2004 Nov 30;2(12):e422. doi: 10.1371/journal.pbio.0020422 (PMC532390; doi:10.1371/journal.pbio.0020422)
Supplement: Table S1 — Also available at http://genes.mit.edu/NielsenEtAl/. (4.3 MB ZIP). [file pbio.0020422.st001.zip › NielsenEtAl/html/240.html]

AN8491.1.NCU00105.1.MG00482.1.FG06962.1


```
 CLUSTAL W (1.82) Multiple Sequence Alignments - Introns Inserted


Sequence 1: MG00482.1	185 aa
Sequence 2: FG06962.1	188 aa
Sequence 3: NCU00105.1	206 aa
Sequence 4: AN8491.1	188 aa
Alignment Length: 211 aa
Number Identitical Residues: 112 aa
Alignment Score (without introns) 5359


MG00482.1 	MRPLTDTETQTLLAK~LANYTGNSLKSLIA~PLED---GDRYVFRLHKDR~VYYVKLSIA
NCU00105.1	MRPLTETEQKTVFEK0LANYCT-DLKSLIA~PLDD---GDRYVFRLNHSR0VYYVRLSVA
FG06962.1 	MRPLTEKETQTLFEK~LANYTGGSLKNLIA~PLDDSPNADRYVFRLVRDR~VFYVRLSIA
AN8491.1  	MRPLTEEETQTLFKK~LAEYCGSGLKELIK1PLDSSPNADRYVFRLSGNR~VYYSLLSIA
          	*****: * :*:: * **:*  ..**.**  **:.:...*******  .* *:*  **:*

MG00482.1 	NLATTIARDKLMSLGTCLG~KFTK0SGKFRLHITAL~PILSEHARYKMWVRSNGEMPFLY
NCU00105.1	NLATSVSRDALLSLGTCLG~KMTK~TGKFRLHITAL1PILSEHARHKIWVKDNGAQPFLY
FG06962.1 	NLATSIAREKLLSLGTCLG1KFTK~TGKFRLHVTAL~PIIAEHARYKIWVKENGTMPFLY
AN8491.1  	NAATAFPRDQLLSLGICMG1KFTK~TGKFRLHITAL~PIIAEHARNKIWIKPNGVMPFLY
          	* **:..*: *:*** *:* *:** :******:*** **::**** *:*:: **  ****

MG00482.1 	GGN-----------------------V1VKAHVGRQTDDCPEHQGIVVYNMQDVPLGFGL
NCU00105.1	GSNGIPLHLLVLCPTSLMKTNGHLKTV~VKAHVGRWSEDCPEHSGVVVYSMADIPLGFGV
FG06962.1 	GSN-----------------------I~VKAHVGRWSEDCPEHQGVVVYSMNDTPLGFGV
AN8491.1  	GSN-----------------------V~AKAHVAKLPDEMAEHSGVIVCTQDDVPLGFGI
          	*.*                       : .****.: .:: .**.*::* .  * *****:

MG00482.1 	TAKSTADARRLDPTGITCFRQSDCGEYLRDEDALFATG
NCU00105.1	TARSTTEARRLDPTGIVCFRQSDCGEYLRDEDTLFAG-
FG06962.1 	TARSTAEARRLDPTGITCFRQADCGEYLRDEDNLFATG
AN8491.1  	TARSTAEARRLDPTGIVCFRQADCGEYLRDEDTLFASG
          	**:**::*********.****:********** *** .
```
